# Proximate Composition, Microbiological Quality and Safety of Raw and Open Sun‐Dried Fish Products in Lake Tana, Ethiopia

**DOI:** 10.1002/fsn3.4671

**Published:** 2024-12-26

**Authors:** Solomon Birie, Minwyelet Mingist, Mulugeta Kibret, Tadlo Yitayew Atlog, Hirut Geremew, Banchiamlak Getnet

**Affiliations:** ^1^ Department of Fisheries and Aquatic Sciences, College of Agriculture and Environmental Sciences Bahir Dar University Bahir Dar Ethiopia; ^2^ Department of Biology, Faculty of Natural and Computational Sciences Debre Tabor University Debre Tabor Ethiopia; ^3^ Department of Biology, College of Sciences Bahir Dar University Bahir Dar Ethiopia; ^4^ Department of Food Engineering, Faculty of Chemical and Food Engineering, Bahir Dar Institute of Technology Bahir Dar University Bahir Dar Ethiopia; ^5^ Department of Animal Production & Technology, College of Agriculture and Environmental Sciences Bahir Dar University Bahir Dar Ethiopia

**Keywords:** fish, Lake Tana, microbial, proximate, quality, safety

## Abstract

The quality and safety of fish products are crucial because poorly handled fish products can result in foodborne illnesses, spoilage, and economic losses. Data on the nutritional and microbiological quality of fish products in Ethiopia, especially in Lake Tana, is limited. This study assessed the proximate composition and microbial quality of raw and open sun‐dried fish products in Lake Tana. Using the standard procedures, 60 fish tissue samples were collected and analyzed for proximate composition and microbial quality. The open sun‐dried fish had significantly higher ash (10.08% ± 1.98%), fat (6.01% ± 1.59%), protein (48.76% ± 8.55%), water activity (0.7358 ± 0.0368) and salt contents (5.89% ± 3.17%) compared to the raw fish (*p* < 0.05). A significant difference was observed between raw and dried fish in aerobic mesophilic bacteria, *Staphylococcus aureus* , yeast and mold counts (*p* < 0.05). The mean total and fecal coliform counts were not significantly different between the raw and dried fish (*p* > 0.05). In raw fish, 16.6% and 83.3% of samples had total coliforms and fecal coliforms exceeding the recommended limits, respectively. Both raw and dried fish samples tested positive for *Salmonella* and *Shigella* spp. There was a strong positive correlation between water activity and microbial load (*r* = 0.756, *p* < 0.05) and also between moisture content and microbial load (*r* = 0.786, *p* < 0.05). Most of the assessed raw and sun‐dried fish samples were above the international microbial specification limits, which need attention to ensure the safety of fish consumers.

## Graphical abstract

This study assessed the nutritional and microbiological quality of raw and open sun‐dried fish products in Lake Tana, Ethiopia. The results showed that open sun‐dried fish had ahigher ash, fat, protein, and salt contents compared to raw fish. There was a significant difference in microbial loads between the two fish products, and most samples were above international microbial specification limits, highlighting the need for attention to ensure consumer safety.

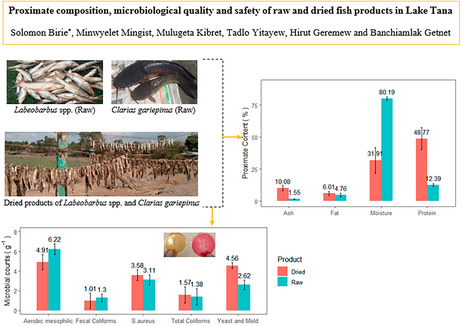

## 1 Introduction

The proximate composition of fish contains moisture, protein, lipid, ash, and carbohydrate contents, which make up around 96%–98% of the tissue (Begum, Akter, and Minar 2012). Fish are an important source of protein, fat, vitamins, and minerals for individuals of all ages (Khalili and Sampels 2018). The protein is high‐quality and easily digestible (FAO 2018), and the fat is the best source of long‐chain omega‐3 polyunsaturated fatty acids (Saini and Keum 2018). But fish's nutritional value differs greatly within and among species, depending on tissue type, habitat, region, and season (Tilami and Sampels 2017; Nerhus et al. 2018). Typically, fish contains approximately 66%–81% moisture, 16%–21% protein, 0.2%–25% fat, and 1.2%–1.5% minerals (Love 1970). Dried fish forms can also provide greater nutritional benefits for many poor individuals worldwide (Kent 2019; Siddhnath Ranjan et al. 2022). In developing countries, traditional fish drying and smoking are common, which account for 12% of all fish destined for human consumption (FAO 2018).

Despite the numerous benefits that fish offer, consumers and regulatory bodies express concerns about nutritional loss, microbial quality, and safety of raw and traditionally dried products (Mithun et al. 2021; Al Banna et al. 2022). As a result, effective preservation and storage methods like refrigeration and drying are needed for easily perishable fish and fish products (Paul et al. 2018; FAO 2022). For instance, improper handling and preservation can lead to microbial spoilage, primarily due to bacteria that invade the tissues from the gills, skin, and intestine of the fish or from microorganisms present in the environment, such as contaminated surfaces (Rathod et al. 2022). High microbial counts, typically exceeding 10^7^ CFU/g, indicate spoilage (Gram and Dalgaard 2002; Sugawara and Nikaido 2014). In addition to spoilage, improper handling of food products at various stages of the value chain can make the products susceptible to microbial and chemical cross‐contamination (Khaliq et al. 2021; Sheng and Wang 2021). Such fish quality defects can be assessed through sensory (smell, taste, and touch), microbial (total aerobic count and detection of pathogens), and chemical methods (Huss, Ababouch, and Gram 2003; Ozogul and Ozogul 2004; Raatikainen et al. 2005). For example, poor sanitary practices during fish handling can be tested using hygiene indicator microorganisms like counts of aerobic mesophilic bacteria, *Staphylococcus aureus* , fecal coliforms, and other bacteria (Sultana et al. 2010; Anihouvi et al. 2019). Microbial contamination of foods of animal origin, including fish, is a serious concern for the consumer that may cause a wide range of foodborne diseases (Zahoor, Liaqat, and Azhar 2018). To address this issue, appropriate preservation methods such as drying, salting, smoking, freezing, canning, and other more recent methods are necessary to extend the product's shelf life (Oparaku and Mgbenka 2012; Abdu et al. 2018; Mei, Ma, and Xie 2019).

In Ethiopia, fish losses are common due to inadequate handling, processing, storage, and preservation practices (Asmare et al. 2015; Tesfay and Teferi 2017; Tut, Wakjira, and Tamire 2019; Deng 2020). This is also true in the case of Lake Tana, where flaws in fish handling, storage, distribution, processing, and marketing have been reported by Mohammed (2011) and Yimer, Mingist, and Bekele (2017). A comprehensive study focusing on the proximate composition, quality, and safety of fishery products in Lake Tana is limited. Of course, Geremew, Abdisa, and Goshu (2020), Gutema and Hailemichael (2021), and Mitiku et al. (2023) have made valuable contributions by investigating specific aspects of these issues in Lake Tana. However, their research is limited in scope, focusing on a small number of landing sites and a restricted set of parameters. This leaves gaps in our understanding of the overall proximate, safety, and quality of the lake's fishery products. As a result, this study was designed to address this gap by assessing the proximate composition, microbial quality, and safety of both raw and open sun‐dried fish products at selected landing sites in Lake Tana.

## 2 Materials and Methods

### 2.1 Description of the Study Area

This study was conducted in Lake Tana, the largest freshwater body in Ethiopia. It is situated at 12° N, 37°15′ E, 1830 altitude, and covers an area of 3050 km^2^ (Vijverberg, Sibbing, and Dejen 2009). The lake is a third‐order lake, with an average depth of 8 m, and its water column does not experience long‐term thermal stratification (Dejen et al. 2004). The area experiences an annual rainfall of up to 2000 mm, with a rainy season from May to October and a peak from July to September (Wondie et al. 2007). The average temperature of the Lake Tana basin is 20°C (Abebe et al. 2017). A total of 27 fish species have been identified and classified into four families: Cyprinidae, Balitoridae, Clariidae, and Cichlidae (Getahun and Dejen 2012; Habteselassie 2012). Many of these fish species are endemic to the lake, and commercially important species targeted for fishing include *Oreochromis niloticus* , *Labeobarbus* species, and *Clarias gariepinus* (Wudneh 1998; Getahun and Dejen 2012). Gill nets are one of the most widely used fishing gear to catch fish. Some fishers also use cast nets, scoop nets, and hooks around Lake Tana. Fish processing is mostly practiced traditionally under non‐hygienic conditions on bare ground for gutting, filleting, or drying with little to no regard for safety (Mengistu et al. 2017). Due to infrastructure limitations like cold storage for fresh fish, open sun‐dried fish is produced in large extent.

### 2.2 Study Design and Sample Collection

**FIGURE 1:**
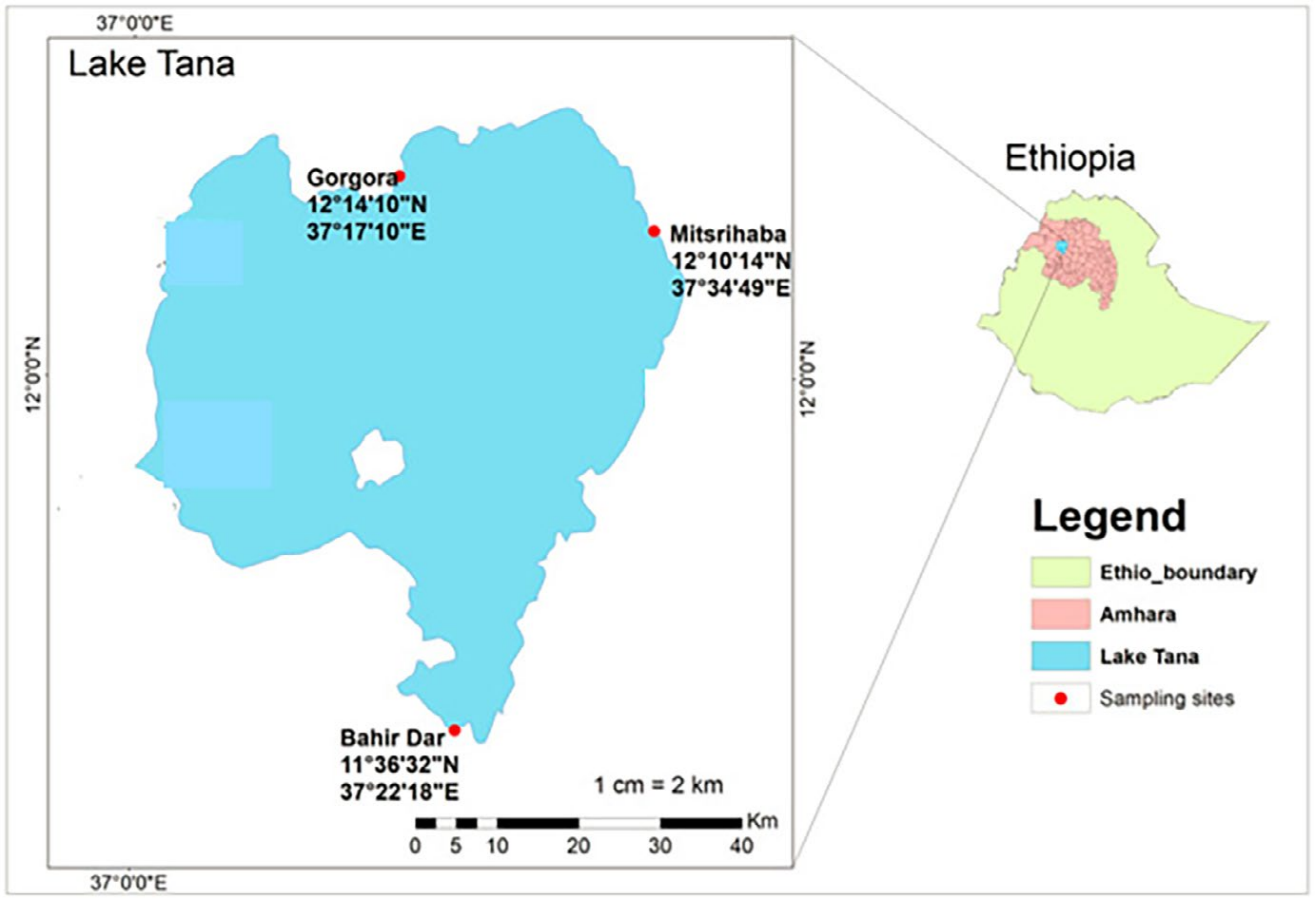
Map of the study area and sampling sites. *Source:* Own map.

The study design was cross‐sectional, and it was conducted during the dry season of the year (January–May, 2023). The sample size to be investigated for the microbial analysis was determined based on sampling guidelines developed by the International Commission on Microbiological Specifications for Foods (ICMSF) (1986). Individual fish were used as a sampling unit, and the appropriate size of the analytical unit was drawn for laboratory analysis. For each individual or sampling unit, appropriate analytical units were used for microbial and proximate analysis. For both fresh and open sun‐dried fish, a total of 60 samples were collected using sterilized utensils in Lake Tana at Bahir Dar, Mitsrihaba, and Gorgora landing sites (Figure 1) in the morning between 8 a.m. and 10 a.m. We selected the three sampling sites as they are the major landing and processing sites, representing the Lake Tana fisheries. For raw and open sun‐dried fish, five individual fish at three landing sites for two species (*Labeobarbus* spp. and *C. gariepinus* ) were collected randomly from the collected mass (10 samples × 3 sites × 2 species = 60). Then, the samples were carefully placed in an ice box (4°C) with labeled plastic bags and transported to Bahir Dar University for analysis.

### 2.3 Proximate Analysis

The proximate composition was determined following the specific instructions outlined in the Association of Official Analytical Chemists (AOAC) manual. The muscle tissues of raw and open sun‐dried fish samples were homogenized by grinding, and the resulting powder was sieved. From the homogenized sample, an appropriate weight was used for assessing the proximate composition (moisture, fat, protein, and ash) and salt content.

### 2.4 Moisture Content

The moisture content was determined using the methods outlined in AOAC (2000). The moisture content was calculated as:

$$\;\text{Moisture}\;\left(\%\right)=\frac{W1-W2}{W1}\;x\;100$$

where, W1 = weight (g) of sample before drying; W2 = weight (g) of sample after drying.

### 2.5 Ash Content

Following the specific instructions outlined in the AOAC (2000) manual. The ash content was calculated as:

$$\mathrm{Ash}\;\left(\%\right)=\frac{\text{Weight of}\;\mathrm{ash}}{\text{Weight of sample}}\;x\;100$$

### 2.6 Crude Protein

The crude protein was determined using the Kjeldahl method, as outlined by AOAC (2000). The total nitrogen and crude protein content in the sample were estimated using the appropriate formula.

$$\%N=\frac{\left(S-B\right)\times M\times 14.01}{\text{Weight of sample}\;}x\;100\;\text{Crude protein}\;\left(\%\right)=\%N\times 6.25$$

where, %N and %P are nitrogen and protein content in percent, respectively; S is the volume of HCI in sample titration; B is the volume of HCI consumed in blank titration; M is the normality of HC1; 14.01 is the molecular weight of nitrogen; 6.25 is a correction factor.

### 2.7 Fat Content

The fat content was determined using the Soxhlet method according to the guidelines provided by the AOAC (2000). The fat content was calculated as:

$$\text{Crude}\;\mathrm{fat}\;\left(\%\right)=\frac{\text{Weight of}\;\mathrm{fat}}{\text{Weight of sample}}\;x\;100$$

### 2.8 Salt Content

The salt content was determined using Mohr's titration method. Then, the salt content was determined using a calculation.

$$\text{NaCl}\%=\frac{\text{Titer value}\times {\text{Normality of AgNO}}_{3}\times 58.4\times 100}{\text{Weight of the sample}\;x\;1000}$$

### 2.9 Water Activity

The water activity of the fish sample was measured using a water activity meter (AQUALAB 4TE, USA, SN: S40003072) at a range of 24°C–27°C.

### 2.10 Microbial Analysis

For microbial analysis, 25 g of the fish fillet were weighted in aseptic conditions inside the biosafety hood. Then, it was mixed with 225 mL of sterilized peptone and a normal saline solution (0.1% peptone and 0.85% NaCl) in a sterilized plastic bag. To achieve homogeneity, the mixture was homogenized for 2 min by using a stomacher*400 circulator (England, SN: 42110). Then, from the homogenized sample, tenfold serial dilutions of 10^−2^, 10^−3^, 10^−4^, 10^−5^, 10^−6^, and 10^−7^ were prepared by adding 1 mL to a sterile test tube containing 9 mL of sterile peptone and normal saline solution, using a sterile pipette. From each serially diluted sample, 1 mL was taken and pour‐plated with sterile 15–20 mL media for enumeration of microbial load (Maturin and Peeler 2001). The Petri dish was, then, inverted and placed in an incubator (DHP‐9052, SN: 0814979) at the appropriate temperature and time. Then, colonies were counted using a colony counter (UK, SN: R000100372) and expressed as the mean colony‐forming units per gram of sample.

### 2.11 Aerobic Mesophilic Count

For the aerobic mesophilic bacteria count, inoculated plate count agar (HiMedia, India) was incubated at 37°C for a duration of 24–48 h. Plates with a range of 30–300 colony‐forming units were enumerated to determine the total load of aerobic mesophilic bacteria (Maturin and Peeler 2001). The recorded result was expressed as the mean colony forming units per gram using the following formula:

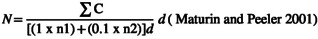

where, *N* = number of colonies per gram; ∑C = sum of counted colonies; n1 = number of plates counted in the first dilution; d = dilution the first count obtained; n2 = number of plates counted in the second dilution.

### 2.12 Yeast and Mold Count

Inoculated acidified potato dextrose agar (HiMedia, India) was incubated at 25°C for 5 days with the addition of 10% tartaric acid to inhibit bacterial growth. After incubation, plates with colony‐forming units ranging from 10 to 150 were counted (Tournas et al. 2001).

#### 2.12.1 *Staphylococcus aureus* Count

Samples inoculated with mannitol salt agar (HiMedia, India) were incubated at 37°C for a period of 24–48 h. Colonies from plates with 20–200 colonies were counted. Representative colonies exhibiting a golden yellow color were selected and then, the catalase and coagulase tests were conducted for confirmation (Tallent et al. 2016).

### 2.13 Total Coliform and Fecal Coliform Counts

The most probable number (MPN) technique was used to enumerate both total and fecal coliforms (Feng et al. 2002). A presumptive, confirmed, and complete test was performed using Lactose Broth (HiMedia, India), 2% Brilliant Green Bile Broth (HiMedia, India), and EC broth (HiMedia, India), respectively. For the presumptive test, each 1 mL of sample was taken from 10^−1^, 10^−2^, and 10^−3^ diluted homogenate and inoculated into three tubes containing 10 mL of lactose broth and an inverted Durham's tube. Following an incubation period of 24–48 h, the number of tubes exhibiting positive gas production was recorded. To confirm the presence of total coliforms, a loopful of broth from each presumptive positive lactose broth tube was gently transferred to tubes containing 10 mL of brilliant green lactose bile 2% broth and an inverted Durham's tube. The tubes were, then, incubated at 37°C for a maximum of 48 h (Feng et al. 2002). For fecal coliform confirmation, a loopful of broth from each presumptive positive lactose broth tube was gently transferred to tubes containing 10 mL of sterilized EC broth and an inverted Durham's tube, which were subsequently incubated at 44.5°C for 24–48 h. Positive tubes identified during the confirmation tests were recorded, and the results were reported using the MPN table (Feng et al. 2002). In the completion stage of the testing process, a loopful of broth from each EC broth tube showing positive results was streaked onto eosin methylene blue agar (HiMedia, India) and incubated at 37°C for 24 h (Feng et al. 2002).

### 2.14 Detection of *Salmonella* and *Shigella* spp.

The homogenized sample was incubated at 37°C for 24 h to enrich the sample with buffered peptone water (HiMedia, India). Then, an inoculum from the enriched culture was streaked on Salmonella and Shigella agar (HiMedia, India) and incubated at 37°C for 24 h. The detection of suspected *Salmonella* and *Shigella* colonies was based on their distinctive appearance on SS agar. The suspected colonies were also subjected to further biochemical tests (Andrews and Jacobson 2013; Andrews et al. 2018).

### 2.15 Data Analysis

The data was analyzed using the Statistical Package for the Social Sciences (IBM SPSS Statistics, Version 26.0). For the microbial counts, the data was transformed and presented as log10 (CFU/g). Descriptive statistics, such as frequency, means, graphs, and tables, were used to visualize the collected data. A one‐way ANOVA was used to compare the mean difference in proximate composition and microbial counts among sites. For comparing the mean difference between the raw and open sun‐dried fish samples, an independent sample *t*‐test was used. The level of significance was set at *p* < 0.05. To indicate significant statistical differences (*p* < 0.05), lowercase superscripts (a–c) were used in the same column of the table for each parameter. In addition, ** indicates significance at 0.01 level, and * is significance at 0.05.

## 3 Results and Discussion

### 3.1 Proximate Composition

#### 3.1.1 Moisture Content

The average moisture content of the raw fish was 80.18% ± 1.43%, and it was not significantly different between the species (*p* > 0.05), as shown in Table 1. However, the moisture content was reduced to 31.91% ± 10.15% for open sun‐dried fish, with a range of 16.7% ± 1.42% to 43.6% ± 2.65% and a significant statistical difference with that of raw fish. This shows that the traditional open sun‐drying method practiced in Lake Tana led to a substantial reduction of approximately 39.53% in the moisture content of raw fish.

**TABLE 1:** Proximate composition (%) of raw and open sun‐dried *Labeobarbus* spp. and *Clarias gariepinus* from different landing sites in Lake Tana (2023).

Parameters	Species	Raw fish (Mean ± SD)	Dried fish (Mean ± SD)
Moisture	*Labeobarbus* spp.	80.57 ± 1.72^a^	31.20 ± 11.89^a^
	*Clarias gariepinus*	79.79 ± 1.01^a^	32.61 ± 8.74^a^
Ash	*Labeobarbus* spp.	1.43 ± 0.15^a^	8.77 ± 1.590^a^
	*Clarias gariepinus*	1.66 ± 0.29^a^	11.38 ± 1.41^b^
Fat	*Labeobarbus* spp.	5.42 ± 1.09^b^	7.29 ± 0.64^b^
	*Clarias gariepinus*	4.08 ± 1.11^a^	4.73 ± 1.12^a^
Protein	*Labeobarbus* spp.	11.89 ± 1.72^a^	51.04 ± 9.28^b^
	*Clarias gariepinus*	12.88 ± 1.06^b^	46.48 ± 7.58^a^
Salt content	*Labeobarbus* spp.	0.07 ± 0.42^a^	5.01 ± 3.11^a^
	*Clarias gariepinus*	0.12 ± 0.05^a^	6.78 ± 3.16^a^
Water activity	*Labeobarbus* spp.	0.99 ± 0.0^a^	0.7302 ± 0.04^a^
	*Clarias gariepinus*	0.99 ± 0.0^a^	0.7415 ± 0.03^a^

*Note:* The superscript lowercase letters (a and b) indicate significant difference at *p* < 0.05 within each column.

The mean moisture content of open sun‐dried fish was statistically different among the three landing sites (*p* < 0.05), with higher values recorded from Mitsrihaba and lower values from the Bahir Dar landing site, as shown in Table 2. The variation might be associated with differences in the drying time, environmental conditions, and salt content used across different landing sites. The findings of this study present a higher moisture content compared to the report by Gutema and Hailemichael (2021) on dried fish products in Ethiopia. These variations may be due to differences in the sampling time of dried fish products, which can be influenced by temperature, wind, and humidity fluctuations during the drying process. According to Samson et al. (2004), dried fish with a moisture content reduced to 25% inhibits bacterial growth, and further reduction to 15% offers good resistance to yeast and mold growth, thereby extending the shelf life. However, in the case of Lake Tana, the moisture content is far from 25%. Such higher moisture content in open sun‐dried fish may have significant implications for product quality, safety, nutritional value, and economic viability. Therefore, reducing the moisture level to the acceptable range using an effective drying process is essential to ensure the production of high quality, safe, and nutritious dried fish products (Gebremedhin et al. 2016).

**TABLE 2:** Proximate composition (%) of raw and open sun‐dried fish samples collected from different landing sites around Lake Tana (2023).

Parameters	Sites	Raw fish (Mean ± SD)	Dried fish (Mean ± SD)
Moisture content	Gorgora	80.74 ± 1.58^a^	34.83 ± 2.06^b^
	Bahir Dar	80.70 ± 1.00^a^	19.10 ± 2.88^a^
	Mitsrihaba	79.11 ± 1.16^a^	41.79 ± 3.75^c^
Ash content	Gorgora	1.57 ± 0.28^a^	9.02 ± 1.72^a^
	Bahir Dar	1.51 ± 0.34^a^	11.54 ± 0.87^b^
	Mitsrihaba	1.56 ± 0.14^a^	9.67 ± 2.34^ab^
Fat content	Gorgora	4.31 ± 0.51^a^	6.32 ± 1.17^a^
	Bahir Dar	3.97 ± 1.11^a^	5.96 ± 2.11^a^
	Mitsrihaba	5.99 ± 1.12^b^	5.74 ± 1.59^a^
Crude protein	Gorgora	12.42 ± 0.84^a^	46.44 ± 4.89^b^
	Bahir Dar	12.62 ± 0.92^a^	59.11 ± 3.27^c^
	Mitsrihaba	12.13 ± 1.40^a^	40.74 ± 1.25^a^
Salt content	Gorgora	0.08 ± 0.24^a^	4.96 ± 1.04^b^
	Bahir Dar	0.10 ± 0.33^a^	2.85 ± 1.10^a^
	Mitsrihaba	0.10 ± 0.08^a^	9.87 ± 1.05^c^
Water activity	Gorgora	0.9901 ± 0.002^a^	0.7595 ± 0.008^b^
	Bahir Dar	0.9909 ± 0.001^a^	0.6876 ± 0.011^a^
	Mitsrihaba	0.9903 ± 0.002^a^	0.7603 ± 0.006^b^

*Note:* The superscript lowercase letters (a, b and c) indicate significant differences at *p* < 0.05 within each column.

#### 3.1.2 Ash Content

The ash content of the raw fish was 1.54% ± 0.25%, and it significantly differed from that of the open sun‐dried fish (10.08% ± 1.98%) (*p* < 0.05). However, there was no statistically significant mean difference among sites for raw fish samples (*p* > 0.05). In the case of open sun‐dried fish, the content varied from 7.75% ± 0.60% to 12.3% ± 0.60%, and statistically significant differences were observed between species, with a higher value recorded for *C. gariepinus* as shown in Table 1. This might be related to the amount of salt added during the drying process or species differences. The findings indicate that the ash content of open sun‐dried fish was higher compared to that of raw fish, which may be attributed to the addition of salt and low moisture content during the drying process. Similar effects of drying and salt addition on ash content were reported by Farid et al. (2016). Additionally, open sun‐dried fish may be exposed to dust particles, which could contribute to an increase in organic matter, as noted by Kumar et al. (2017).

#### 3.1.3 Fat Content

The fat content of the raw fish was 4.75% ± 1.27%, and it significantly differed from that of the open sun‐dried fish sample, which was 6.01% ± 1.59% (*p* < 0.05). This was higher than that of the Geremew, Abdisa, and Goshu (2020) report, and the difference might be associated with exogenous and endogenous factors like the season of sampling, geographical sites, diets, and nutrient digestibility (Taşbozan and Gokçe 2017). There were statistically significant differences in the fat content of raw fish between the species and among sites (*p* < 0.05), with a higher mean value at the Bahir Dar site. As shown in Table 1, the fat content of open sun‐dried fish ranged from 4.10% ± 0.58% to 7.84% ± 0.57%, and a statistically significant difference was observed between species (*p* < 0.05), with dried *Labeobarbus* spp. showing a higher fat content compared to dried *C. gariepinus* . The observed variations in fat content might be due to variations in diet composition, species‐specific physiological characteristics, and the body weight of the fish (Farid et al. 2014; Naqvi et al. 2021).

#### 3.1.4 Crude Protein Content

The average protein content of traditionally open sun‐dried fish was 48.76% ± 8.55%, ranging from 49.1% ± 1.75% to 63.2% ± 0.525%, and significantly higher than the mean value of raw fish, which was 12.39% ± 1.04% (*p* < 0.05). There was no statistically significant mean difference among sites for raw fish samples (*p* > 0.05), but it was significant for open sun‐dried fish samples (*p* < 0.05) as shown in Table 2. According to Haque (2004), sun‐dried fish can contain up to 80% protein, but the protein content of dried fish products from Lake Tana was low. This could be due to factors such as poor drying methods, the use of salt during drying, the type of fish species, and food availability. Different studies have shown that the addition of salt during drying (Kumar et al. 2017) and longer drying time (Guinee 2021) probably lead to lower protein content in dried fish products. However, the findings of this study indicate that dried fish products from Lake Tana still serve as a valuable source of protein compared to raw fish.

#### 3.1.5 Water Activity and Salt Content

The average water activity of the raw fish was 0.99% ± 0.0%, and it significantly differed from that of the open sun‐dried fish samples, which was 0.7358% ± 0.0368% (*p* < 0.05). Lower water activity in open sun‐dried fish suggests the less likely it was exposed for microorganisms to grow and spoil the product. The average salt content was also significantly higher in the dried fish sample (5.89% ± 3.17%) compared to the raw fish sample (0.10% ± 0.05%). The addition of salt during the open sun‐drying process is common around Lake Tana; this might be the reason for the higher salt content in the dried fish product. A Pearson correlation coefficient was computed to determine if there is a relationship between the microbial load and water activity, moisture, and salt content of the fish product sample. There was a strong positive correlation between water activity and microbial load (*r* = 0.756, *p* < 0.05) and between moisture content and microbial load (*r* = 0.786, *p* < 0.05), as shown in Table 3. Increases in water activity and moisture content were correlated with increases in microbial load.

**TABLE 3:** Correlation matrix showing Pearson's coefficient (*r*) and the level of significance between variables (2023).

Variables	Microbial load	Water activity	Moisture content	Salt content
Microbial load	1			
Water activity	0.756**	1		
Moisture content	0.786**	0.993**	1	
Salt content	−0.289	−0.701*	−0.628*	1

*Note:* *p < 0.05; **p < 0.01.

### 3.2 Microbial Quality and Safety Analysis

#### 3.2.1 Aerobic Mesophilic Bacteria Count

An independent sample *t*‐test revealed a significant statistical difference in aerobic mesophilic bacteria count between the raw and open sun‐dried fish samples (*p* < 0.05). The average aerobic mesophilic bacteria load in raw fish was 6.21 ± 0.56 log CFU/g, ranging from 4.66 to 6.71 log CFU/g, as shown in Table 4. This was higher than that of Mitiku et al. (2023) in the same lake, Onjong et al. (2018) in Kenya, and Quaiyum et al. (2013) in Bangladesh. However, it was comparable to those reported in Egypt by Eltholth et al. (2018) and in Bangladesh by Nur, Ghosh, and Acharjee (2020). The load variation could be due to a number of factors, including the type of fish examined, the addition of preservatives (Aziz and Karboune 2018), and the handling and storage practices (Turner, Luo, and Buchanan 2020).

**TABLE 4:** The mean aerobic mesophilic bacteria, *Staphylococcus aureus* , and yeast and mold count of raw and open sun‐dried *Labeobarbus* spp. and *Clarias gariepinus* in Lake Tana (2023).

Loads	Products	Fish species	*n*	Mean ± SD (current study)	Acceptable limits (International Commission on Microbiological Specifications for Foods (ICMSF) 1986)
Aerobic mesophilic count	Raw fish	*Labeobarbus* spp.	15	6.46 ± 0.74^b^	< 5.5
		*Clarias gariepinus*	15	5.95 ± 0.71^a^	
	Dried fish	*Labeobarbus* spp.	15	4.56 ± 0.80^a^	< 5.5
		*Clarias gariepinus*	15	5.24 ± 0.55^b^	
*Staphylococcus aureus* count	Raw fish	*Labeobarbus* spp.	15	3.31 ± 0.36^b^	< 3
		*Clarias gariepinus*	15	2.91 ± 0.61^a^	
	Dried fish	*Labeobarbus* spp.	15	3.70 ± 0.56^a^	< 3
		*Clarias gariepinus*	15	3.46 ± 0.50^a^	
Yeast and Mold count	Raw fish	*Labeobarbus* spp.	15	2.96 ± 0.46^b^	< 3
		*Clarias gariepinus*	15	2.28 ± 0.10^a^	
	Dried fish	*Labeobarbus* spp.	15	4.38 ± 0.16^a^	< 3
		*Clarias gariepinus*	15	4.72 ± 0.31^b^	

*Note:* The superscript lowercase letters (a and b) indicate significant difference at *p* < 0.05 within each column.

Abbreviation: *n* = number of samples.

For open sun‐dried fish, the average aerobic mesophilic bacteria load was 4.90 ± 0.76 log CFU/g, ranging from 3.49 to 5.98 log CFU/g, of which 23.3% exceeded the acceptable limit set by the International Commission on Microbiological Specifications for Foods (ICMSF) (1986). The lower count in an open sun‐dried fish compared to the raw might be associated with lower moisture content, lower water activity, and higher salt content, as indicated in Table 1. The mean load in this study was lower than that of the Gutema and Hailemichael (2021) report in Ethiopia; it might be due to drying time, water activity, moisture content, storage conditions, and handling practices (Hammond et al. 2015).

**FIGURE 2:**
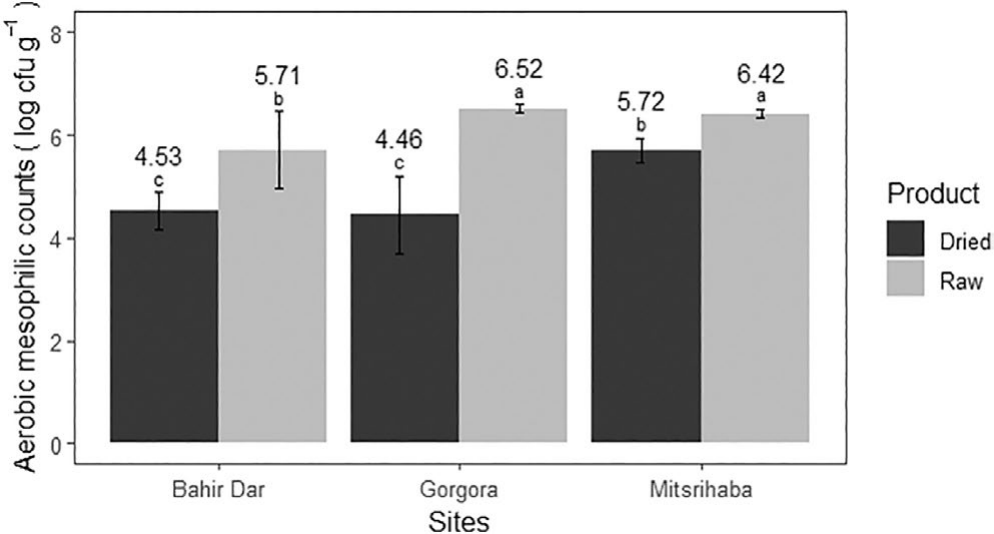
The aerobic mesophilic bacteria count per gram of raw and open sun‐dried fish samples at three landing sites in Lake Tana (2023).

Regarding the landing sites, almost similar circumstances were observed at the three landing sites in both raw and open sun‐dried fish samples as shown in Figure 2. This suggests a general lack of proper hygiene and sanitation practices in fish handling and processing around Lake Tana. However, at Mitsrihaba landing site, the mean load of open sun‐dried fish was somewhat higher than that of Bahir Dar and Gorgora sites. This might be related to the higher moisture content of the product as shown in Table 2. Most of the raw fish samples (86.6%) exceeded the acceptable limit set by the International Commission on Microbiological Specifications for Foods (ICMSF) (1986). Such a high aerobic mesophilic bacteria count in raw fish suggests poor water quality, handling practices, and exposure to unhygienic conditions, which might cause quick spoilage, financial losses, and health risks for consumers. Due to the high microbial load, the possibility of introducing pathogenic bacteria into the fish may be high, leading to quick quality deterioration and food poisoning. The higher microbial counts in raw and dried fish products revealed the need for hygienic handling of these products in accordance with the Codex Alimentarius International Food Standards, specifically with the principles of food hygiene (CXC 1–1969) and the code of practice for fish and fishery products (CXC 52–2003). Therefore, addressing the current hygiene condition of fish handlers around Lake Tana through awareness creation on the proper and hygienic handling of fish is needed to ensure quality and safety of the products.

#### 3.2.2 *Staphylococcus aureus* Count

**FIGURE 3:**
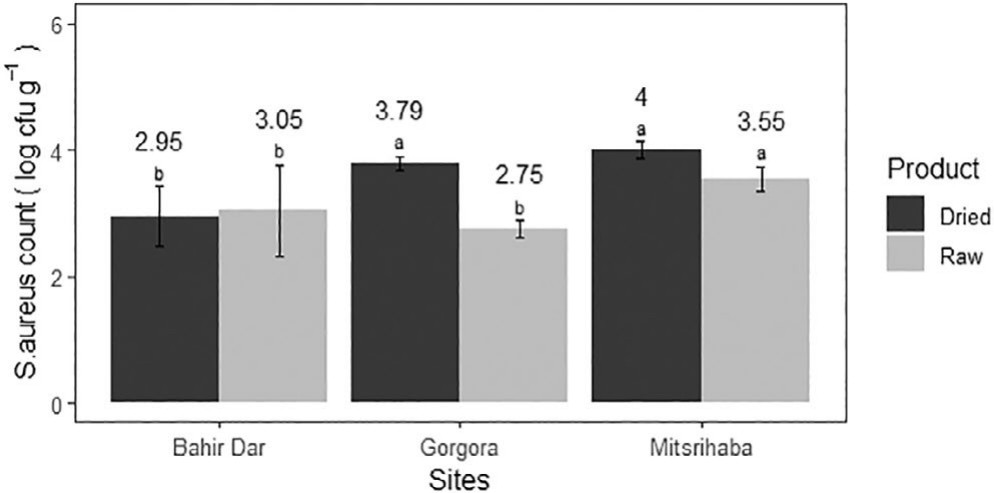
The *Staphylococcus aureus* count per gram of raw and open sun‐dried fish samples at three landing sites in Lake Tana (2023).

The average load of *S. aureus* in raw fish was 3.11 ± 0.53 log CFU/g, with a maximum of 3.84 log CFU/g and a minimum of 2.12 log CFU/g. In the case of open sun‐dried fish samples, the mean load was 3.58 ± 0.53 log CFU/g, ranging from 2.36 to 4.14 log CFU/g. Significant differences were observed between the mean *S. aureus* count of raw and open sun‐dried fish samples (*p* < 0.05). The mean count difference among sites is indicated in Figure 3.

From the analyzed samples, 50.0% of raw and 76.6% of open sun‐dried fish samples exceeded the acceptable limit set by International Commission on Microbiological Specifications for Foods (ICMSF) (1986). This finding was higher than that of Mitiku et al. (2023) and lower than that of Gutema and Hailemichael (2021). However, it was comparable to the report of Eltholth et al. (2018) from Egypt. The variation might be due to the different situations of fish handlers at different times and fishing conditions. The higher count of this bacteria in both samples indicates cross‐contamination, which might be due to poor handling and hygiene practices during the handling process (Simon and Sanjeev 2007); this could also affect consumer confidence in the safety of fish products from Lake Tana. According to Liaqat et al. (2019), food safety can be maintained through good handling practices and proper cooking. However, since *S. aureus* can produce toxins that might survive even after cooking, focusing on safe handling and processing using clean, sanitized equipment and good personal hygiene are important in preventing cross‐contamination and ensuring the safety of raw and open sun‐dried fish. Implementing regular monitoring on the quality and safety of fish products is also highly recommended.

#### 3.2.3 Yeast and Mold Count

**FIGURE 4:**
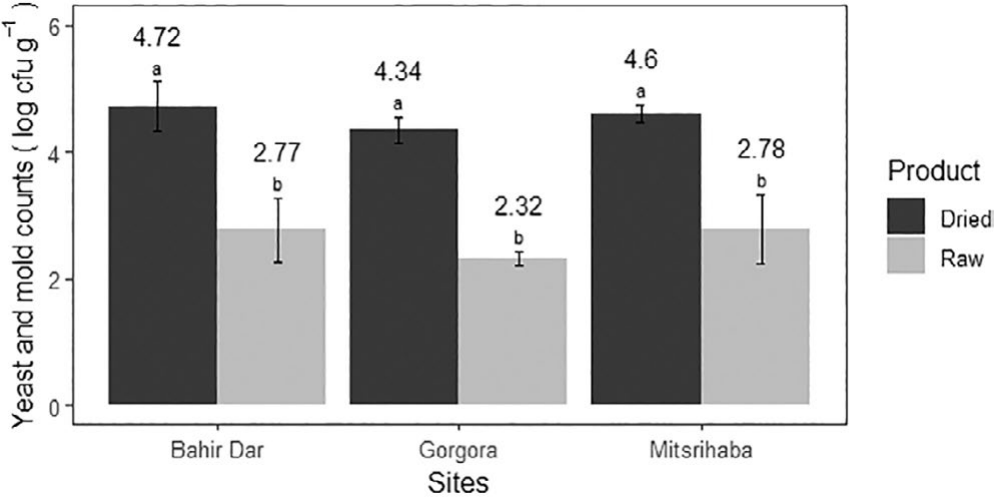
The yeast and mold count per gram of raw and open sun‐dried fish samples at three landing sites in Lake Tana (2023).

A significant statistical difference in yeast and mold counts was observed between the raw and open sun‐dried fish samples (*p* < 0.05). The mean yeast and mold count for raw fish was 2.62 ± 0.47 log CFU/g, which was lower than the mean count of open sun‐dried fish samples (4.55 ± 0.30 log CFU/g). No statistical difference was found among sites in the mean yeast and mold counts for both raw and open sun‐dried fish samples (*p* > 0.05) as shown in Figure 4.

A higher count of open sun‐dried fish samples compared to raw fish might be related to the drying process, where the fish became more exposed to contamination for a longer period. Such a higher load might cause spoilage and reduced shelf life, leading to off‐flavors, odors, and changes in texture. All the examined open sun‐dried fish samples exceeded the acceptable limit set by the European Food Safety Authority (EFSA) 2012. A similar finding was reported by Gutema and Hailemichael (2021). This might be due to poor handling practices during the drying process and poor storage conditions (Moosavi‐Nasab, Asgari, and Oliyaei 2019). Therefore, it is crucial to ensure the safety and quality of raw and dry fish through proper handling, processing, and storage to prevent the risk of foodborne illnesses and economic losses.

#### 3.2.4 Total and Fecal Coliform Counts

The mean total and fecal coliform of raw fish samples were 1.38 ± 0.84 and 1.29 ± 0.39 log MPN/g, respectively, as shown in Table 5. Findings from this study indicate that 16.6% exceeded the recommended limits for total coliforms and 83.3% exceeded the recommended limits for fecal coliforms in raw fish. Comparable results were also reported from different countries. For example, in Kenya, Onjong et al. (2018) found the total and fecal coliform counts exceeded the recommended limit for raw fish samples. In Egypt, Eltholth et al. (2018) also reported a higher load of *E. coli* (10^3^ CFU/g) from fresh tilapia. Conversely, our study result was comparable with that of Quaiyum et al. (2013) report in Bangladesh, which was 36.00 ± 2.33 MPN/g for total coliforms and < 6.2 MPN/g for fecal coliforms for raw fish samples. Nuwanthi et al. (2016) in Sri Lanka also reported a comparable count of coliform (4 MPN/g) in raw fish.

**TABLE 5:** Total and fecal coliform counts of raw and open sun‐dried *Labeobarbus* spp. and *Clarias gariepinus* fish samples in Lake Tana (2023).

Microbial groups	Products	Species	*n*	Mean ± SD (current study)	Acceptable limits (International Commission on Microbiological Specifications for Foods (ICMSF) 1986)
Total coliforms	Raw fish	*Labeobarbus* spp.	15	1.77 ± 0.95^b^	< 2
		*Clarias gariepinus*	15	0.99 ± 0.48^a^	
	Dried fish	*Labeobarbus* spp.	15	1.34 ± 0.71^a^	< 2
		*Clarias gariepinus*	15	1.79 ± 0.92^a^	
Fecal coliforms	Raw fish	*Labeobarbus* spp.	15	1.51 ± 0.19^b^	< 1.04
		*Clarias gariepinus*	15	1.07 ± 0.43^a^	
	Dried fish	*Labeobarbus* spp.	15	0.83 ± 0.52^a^	< 1.04
		*Clarias gariepinus*	15	1.13 ± 0.96^a^	

*Note:* The superscript lowercase letters (a and b) indicate significant difference at *p* < 0.05 within each column.

Abbreviation: *n* = number of samples.

**FIGURE 5:**
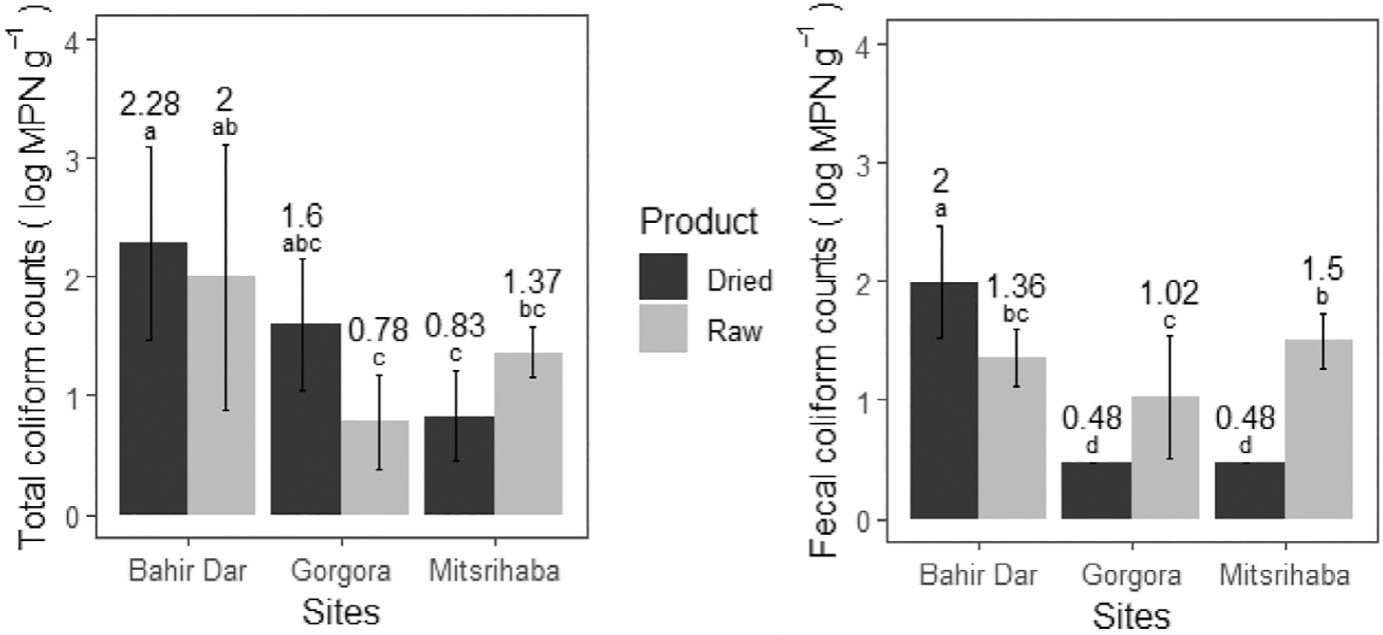
Total and fecal coliform counts per gram of raw and open sun‐dried fish samples at three landing sites in Lake Tana (2023).

For open sun‐dried fish, the mean total and fecal coliform were 1.57 ± 0.84 log MPN/g and 0.98 ± 0.77 log MPN/g, respectively, which did not significantly differ from the mean count of raw fish samples (*p* > 0.05). However, there was a significant mean difference in fecal coliform counts among sites (*p* < 0.05), with the highest count recorded at the Bahir Dar landing site, as shown in Figure 5. The variation among the sites might be due to differences in anthropogenic activities and exposure to waste discharge. Different reports indicated that the Lake Tana water quality around Bahir Dar landing site (Gulf of Lake Tana) was polluted with fecal coliforms (Yimenu 2005; Sitotaw et al. 2022), in which 49% of the nearby population use traditional and poorly constructed latrines (Yimenu 2005). Goshu et al. (2010) also reported that the southern Gulf of Lake Tana is receiving excessive potential anthropogenic point source pollution. The findings from open sun‐dried fish indicated that 23.3% and 33.3% of the sample exceeded the recommended limits for total coliforms and fecal coliforms, respectively.

The presence of total and fecal coliforms in raw and dried fish may indicate poor hygiene and sanitation practices during the processing and handling of fish. Contaminated water sources due to the discharge of untreated sewage, agricultural run‐off, and industrial waste might also cause cross‐contamination. For instance, Goshu et al. (2010) reported fecal contamination in Lake Tana water, which could lead to cross‐contamination of fish. This highlights the need for proper sanitation and hygienic practices during processing and handling to ensure the safety of consumers. In addition, the raw and dried fish products should comply with microbiological criteria established in accordance with the principles and guidelines for the establishment and application of microbiological criteria related to foods (CXG 21–1997). Therefore, empowering fish handlers towards food safety practices like the use of gloves, proper handwashing, cutting fingernails, sanitizing processing materials, and creating environmental hygiene might reduce the bacterial cross‐contamination of the fish product. In addition, multidimensional analytical investigation on the source of fecal coliform contamination is essential.

#### 3.2.5 *Salmonella* and *Shigella* spp.

From the total 30 raw fish samples examined at 3 landing sites in Lake Tana, it was found that all of them tested positive for both *Salmonella* and *Shigella* spp. Similarly, out of the total 30 open sun‐dried fish samples examined at the same landing sites, 33.3% and 66.6% tested positive for *Salmonella* and *Shigella* spp., respectively. In contrast, Mitiku et al. (2023) reported lower prevalence (6% for *Salmonella* and 2.4% for *Shigella*) in the same lake (Ethiopia). The variation might be related to the product type examined, season of sampling, and landing site differences (Atwill and Jeamsripong 2021). These findings indicate the potential risk of *Salmonella* and *Shigella* spp. contamination in raw and dried fish that might be sourced from poor hygiene practices and unsanitary conditions during fishing, processing, and storage. This might be due to most fishers, processors, and traders handling fish under unhygienic conditions with no basic processing facilities in Ethiopia (Bedane, Agga, and Gutema 2022) and specifically in Lake Tana (Mengistu et al. 2017). Thus, proper handling during processing and cooking during consumption is highly recommended to ensure consumers' health and safety. Further investigation on the source of contamination, analyzing the current fish handling practices, and continuous monitoring are essential.

## 4 Conclusions

In conclusion, as per the International Commission for Microbiological Specifications for Food, most of the raw and open sun‐dried fish samples of Lake Tana were above the recommended limits. This may indicate a lack of proper hygiene and sanitation practices in both raw and open sun‐dried fish handling and processing at the three landing sites, especially at the Bahir Dar landing site, where higher coliform counts were recorded. The higher level of fecal bacteria counts and the detection of *Salmonella* and *Shigella* spp. in the fish samples are serious public health concerns that indicate fecal contamination. The higher bacteria load can lead to fish spoilage and make the fish less acceptable for consumption. Generally, the microbial counts of our study revealed an unsatisfactory level of raw and dried fish quality and safety, which needs improvement through the implementation of national and international food safety standards and regulations. Thus, the trade practice and consumers' protection proclamation 685/2010 of Ethiopia should contribute towards achieving better results in food safety assurance of fish products. The traditional open sun‐drying method practiced in the study sites could not achieve the desired moisture level, which needs more effort to reduce further for better nutritional value, microbial quality, and prolonged shelf life, especially at Gorgora and Mitsrihaba landing sites. Therefore, practicing easy and locally available alternative drying methods like solar tent drying is recommended to get better prices and to produce safe and high‐quality dried fish products with enhanced nutritional value. Furthermore, a detailed study should be conducted to confirm the quality and safety of all aspects of the fish production process, such as the fishing nets, boats, fishing holding materials, landing sites, processing and drying materials, processors, and other issues.

## Author Contributions

**Solomon Birie:** conceptualization (equal), formal analysis (equal), investigation (equal), methodology (equal), writing – review and editing (equal). **Minwyelet Mingist:** conceptualization (equal), supervision (equal), visualization (equal), writing – review and editing (equal). **Mulugeta Kibret:** conceptualization (equal), visualization (equal), writing – review and editing (equal). **Tadlo Yitayew Atlog:** investigation (equal), writing – review and editing (equal). **Hirut Geremew:** investigation (equal), writing – review and editing (equal). **Banchiamlak Getnet:** investigation (equal), writing – review and editing (equal).

## Ethics Statement

The authors have collected fish tissue samples from the fish caught by fishers, and we certify that this study followed all the applicable guidelines for the care and use of fish.

## Conflicts of Interest

The authors declare no conflicts of interest.

## Data Availability

The authors declare that data are available from the corresponding author upon request.
